# *Streptococcus thermophilus* iHA318 Improves Dry Eye Symptoms by Mitigating Ocular Surface Damage in a Mouse Model

**DOI:** 10.3390/microorganisms12071306

**Published:** 2024-06-27

**Authors:** Yu-Wei Chang, Yen-Ling Sun, Evelyn Chu, Yi-Yun Hung, Wei-Chieh Liao, Su-Min Tsai, Tsung-Han Lu, Pin-Chao Huang, Chin-Hsiu Yu, Shao-Yu Lee, Han-Hsin Chang, David Pei-Cheng Lin

**Affiliations:** 1 Department of Medical Laboratory and Biotechnology, Chung Shan Medical University, Taichung 40201, Taiwan; golden3p@csmu.edu.tw (Y.-W.C.); lilliansun0429@gmail.com (Y.-L.S.); evelyn20040504@gmail.com (E.C.); jennyhung0820@gmail.com (Y.-Y.H.); a0966150629@gmail.com (W.-C.L.); g99231001@gmail.com (S.-M.T.); 2 Institute of Medicine, Chung Shan Medical University, Taichung 40201, Taiwan; rightlu1114@gmail.com; 3 Percheron Bioceutical Co., Ltd., Taichung 40201, Taiwan; stanley.huang@nutrarex.com.tw (P.-C.H.); yuna.yu@nutrarex.com.tw (C.-H.Y.); shaoyu.lee@nutrarex.com.tw (S.-Y.L.); 4 Department of Nutrition, Chung Shan Medical University, Taichung 40201, Taiwan; jhhc@csmu.edu.tw; 5 Department of Ophthalmology, Chung Shan Medical University Hospital, Taichung 40201, Taiwan

**Keywords:** *Streptococcus thermophilus*, probiotics, dry eye symptoms, ocular surface damage, tear volume, tear film breakup time, tear film stability, mouse model

## Abstract

Dry eye is a complicated ocular surface disease that causes discomfort, visual disturbance, and frequently observed ocular surface damage. Emerging hypotheses suggest probiotics may help relieve dry eye symptoms by modulating inflammation and oxidative stress. This study aimed to investigate the therapeutic effects of *Streptococcus thermophilus* iHA318 probiotics on dry eye using in vitro assays and an in vivo murine model of ultraviolet B (UVB) radiation-induced dry eye. In vitro analyses revealed that *S. thermophilus* iHA318^®^ exhibited antioxidant activity and anti-inflammatory effects by inhibiting reactive oxygen species production and suppressing inflammatory cytokines. For the in vivo study, female ICR mice were assigned to normal control, UVB-induced dry eye, and UVB+iHA318 treatment groups. UVB exposure significantly decreased tear volume and tear film breakup time (TBUT) compared to normal controls. Supplementation with *S. thermophilus* iHA318^®^ via oral gavage markedly improved tear production and TBUT on day 7 post-UVB exposure. Ocular surface photography demonstrated improved gradings of corneal opacity, smoothness, and lissamine green staining in the iHA318 group versus the UVB group. Topographical analysis further revealed improvement in the UVB-induced corneal irregularities by iHA318 treatment. Collectively, these results indicate that *S. thermophilus* iHA318 exerts a protective effect against dry eye symptoms by mitigating oxidative stress and inflammation, thereby preserving tear film stability and ocular surface integrity. This probiotic strain represents a promising therapeutic approach for managing dry eye syndrome.

## 1. Introduction

Dry eye is one of the most prevalent ocular surface diseases, affecting millions of people worldwide. It is characterized by a deficiency in tear quality or quantity, leading to ocular discomfort, visual disturbances, and frequently observed ocular surface damage [1]. The pathogenesis of dry eye is multifactorial, with inflammation and oxidative stress playing central roles [2,3]. A chronic inflammation status disrupts tear film homeostasis and promotes ocular surface damage, while oxidative stress exacerbates inflammatory activities and cellular injury [4].

In recent years, probiotics have emerged as a promising therapeutic approach for managing dry eye disease, hypothetically due to their immunomodulatory effects [5]. Probiotics are live microorganisms that confer health benefits when consumed adequately [6]. Their mechanisms of action involve modulating the gut microbiota composition and metabolic activities, which can influence systemic inflammatory and oxidative stress responses [7,8]. The gut microbiota plays a crucial role in regulating immune homeostasis through its interactions with the host’s immune system. Dysbiosis, or an imbalance in the gut microbial communities, can lead to excessive inflammation and impaired immunoregulatory mechanisms [9,10]. Probiotics help restore this balance by promoting the growth of beneficial microbes, producing anti-inflammatory metabolites, and interacting with immune cells [11]. For instance, certain probiotic strains have been shown to induce the differentiation of regulatory T cells (Tregs), which suppresses inflammatory responses [12].

Additionally, probiotics can counteract oxidative stress, a key pathogenic factor in dry eye [13]. Some probiotic strains can produce enzymes that scavenge reactive oxygen species (ROS) or enhance the host’s endogenous antioxidant defense mechanisms [14]. By reducing oxidative stress, probiotics may potentially protect ocular surface cells from damage and attenuate inflammatory cascades.

Some bacteria strains produce exopolysaccharides (EPS) during fermentation [15]. Hyaluronic acid represents one of the EPS and benefits human health [16]. Hyaluronic acid can strongly bond water to tissues, preventing dehydration and providing an important lubricant for the eyes and joints. Also, hyaluronic acid is a strong antioxidant that eliminates ROS stress [17]. Several studies have indicated that hyaluronic acid significantly improves dry eye syndrome by increasing the hydration of the eyes [18,19].

Furthermore, sialic acids are a group of 9-carbon carboxylated monosaccharides with properties that include anti-inflammation, improving cognitive ability, and being indispensable for infant brain development [20,21,22]. A previous study has suggested that a specific strain of *Lactobacillus plantarum* is able to produce sialic acid and present it on the surface of the bacteria. This finding raises the potential of utilizing probiotics as a supplier of sialic acid [23]. However, the research remains limited and requires further investigation.

Previous studies have demonstrated the therapeutic potential of various probiotic strains in alleviating dry eye symptoms and improving ocular surface parameters [24,25]. However, the specific effects of *Streptococcus thermophilus* iHA318^®^, a probiotic strain with reported antioxidant and anti-inflammatory properties, as well as the ability to generate large amounts of hyaluronic acid and sialic acid, on dry eye disease have not been explored. This study aimed to investigate the therapeutic efficacy of *S. thermophilus* iHA318^®^ in an in vivo dry eye model induced by ultraviolet B (UVB) radiation and elucidate its underlying mechanisms through in vitro assays.

## 2. Materials and Methods

### 2.1. Bacterial Activation and Culture

The cryovial containing Streptococcus thermophilus iHA318 was thawed and inoculated into MRS (De Man, Rogosa and Sharpe) medium at a concentration of 1%. Incubation was carried out at 37 °C for 16–24 h in an anaerobic environment to activate the iHA318 strain. Subsequently, the activated bacterial solution was transferred into fresh MRS medium and cultivated anaerobically at 37 °C for an additional 16–24 h.

### 2.2. Bacterial Identification

The identification of the *Streptococcus thermophilus* iHA318 strain was performed using the API^®^ 50 CHL microbial identification kit (BioMerieux, Marcy-l’Étoile, France), along with conventional methods for extracting total RNA. Reverse transcription was performed, and the 16S rDNA served as the target gene for bacterial identification. Three sets of different universal primers were employed for polymerase chain reaction (PCR) amplification of the 16S rDNA [26,27,28] (Table 1). The obtained nucleic acid fragment was compared with the 16S rRNA gene sequences of other strains of *Streptococcus thermophilus* in the GenBank database. Sequence analysis revealed more than 99% similarity, confirming the strain as *Streptococcus thermophilus*.

### 2.3. Cell Culture and Treatment

The murine macrophage cell line RAW 264.7 was obtained from the Bioresource Collection and Research Center (BCRC, Taiwan). The cells were cultured in Dulbecco’s Modified Eagle Medium (DMEM, Gibco) supplemented with 10% fetal bovine serum (FBS, Gibco) and an antibiotic–antimycotic solution containing 100 U/mL penicillin, 100 μg/mL streptomycin, and 0.25 μg/mL amphotericin B. For the experiments, RAW264.7 cells were seeded at 1 × 10^4^ cells/well in 96-well plates with 100 μL medium per well and incubated for 24 h at 37 °C. AAPH was used as an oxidant to induce oxidative stress, while LPS was used as a pro-inflammatory agent to stimulate an inflammatory response in the cells. For the antioxidant assay, the study groups were as follows: (1) Control: medium only; (2) comparative: medium without pre-treatment, then 60 mM AAPH (2,2′-Azobis(2-amidinopropane) dihydrochloride, sigma) for 24 h; (3) experimental: iHA318 solution for 24 h, followed by 60 mM AAPH for 24 h. For the anti-inflammatory assay, the study groups were as follows: (1) Control: medium only; (2) comparative: medium for 24 h, then 1 μg/mL LPS (Sigma, MO, United States) for 48 h; (3) experimental: iHA318 solution for 24 h, followed by 1 μg/mL LPS for 48 h.

### 2.4. NO Production Assay for Assessment of Inflammatory Response

From each experimental group, 50 μL of cell supernatant was collected, and 50 μL of Griess reagent was added to react for 15 min. The absorbance at 550 nm of each group was measured to quantify the concentration of nitric oxide secreted by RAW264.7 cells, indicating the stimulated inflammatory response. The results were presented by the nitric oxide concentration out of the control group set as 100%, while the data from other groups were normalized to the control group.

### 2.5. Osmolality Damage Test

ARPE-19 cells (BCRC, Taiwan), human retinal pigment epithelium cells, were seeded in a 96-well plate at a density of 8.0 × 10^3^ cells per well in 100 μL of DMEM/Ham’s F12 medium supplemented with 10% FBS and an antibiotics cocktail (Gibco). The plate was then incubated at 37 °C in a 5% CO_2_ atmosphere for 24 h. Following cell attachment, test samples were prepared by dissolving them in a culture medium with or without 160 mM NaCl (Sigma). After removing the original cell culture supernatant, 200 μL of the sample solution was added to each well of the 96-well plate containing the ARPE-19 cells. The cells were then co-incubated for 4 h, and then cell viability was determined using the MTT assay.

### 2.6. MTT Assay for Cell Viability

After treatment with the specified concentrations of *S. thermophilus* iHA318 or culture medium (control) with or without stimulus (NaCl or AAPH) for the indicated time periods, the cell culture medium was replaced with 100 μL of MTT reagent (Gibco) and incubated at 37 °C for 2 h. Subsequently, the MTT reagent was aspirated, and 100 μL of dimethyl sulfoxide (DMSO, Sigma) was added to each well for 10 min to dissolve the formazan crystals. The absorbance of each group at 570 nm was measured using a microplate reader to assess cell viability, with the control group normalized to 100%.

### 2.7. ROS Production Analysis

After ARPE-19 cell attachment, the medium was replaced with 2 mL of test sample solutions and co-incubated for 24 h at 37 °C. Cells were then detached using trypsin-EDTA, collected by centrifugation, and the pellets were resuspended. Cellular ROS levels were detected using a ROS assay kit (Cayman, Product No.601520). Briefly, the cells were incubated with DCFDA dye for 45 min; then fluorescence was measured (Ex 485 nm/Em 520 nm) after adding the assay buffer.

### 2.8. Quantification of Hyaluronic Acid

Hyaluronic acid production by *S. thermophilus* iHA318 was quantified using a colorimetric method. Briefly, samples were precipitated with 95% ethanol and centrifuged to remove supernatants. The hyaluronic acid precipitate was dissolved in water and depolymerized by heating with borax–sulfuric acid solution at 100 °C for 10 min. After cooling and adding carbazole reagent, the mixture was reheated at 100 °C for 15 min for color development. Aliquots (100 μL) of the color development reactions were transferred to a 96-well plate, and the absorbance was measured at 525 nm. Concentrations were determined using a glucuronic acid standard curve with a 2.07 correction factor to account for molecular weights.

### 2.9. Quantification of Sialic Acid

The thiobarbituric acid assay was applied to quantify the sialic acid content [29]. Samples were hydrolyzed with 8 N HCl at 80 °C for 2 h, then neutralized with 8 N NaOH. The hydrolyzed samples were mixed with 0.2 M NaIO4 (dissolved in 9 M H3PO4) and left to react at room temperature for 20 min to oxidize sialic acid. The reaction was stopped with an arsenite solution. The thiobarbituric acid reagent was added and heated at 100 °C for 15 min to form the sialic acid–thiobarbituric acid complex. After cooling and phase separation using cyclohexanone and centrifugation, absorbance at 549 nm was measured to determine sialic acid concentrations according to the standard curve with N-acetylneuraminic acid.

### 2.10. UVB-Induced Mouse Dry Eye Model and Treatment

**Figure 1:**
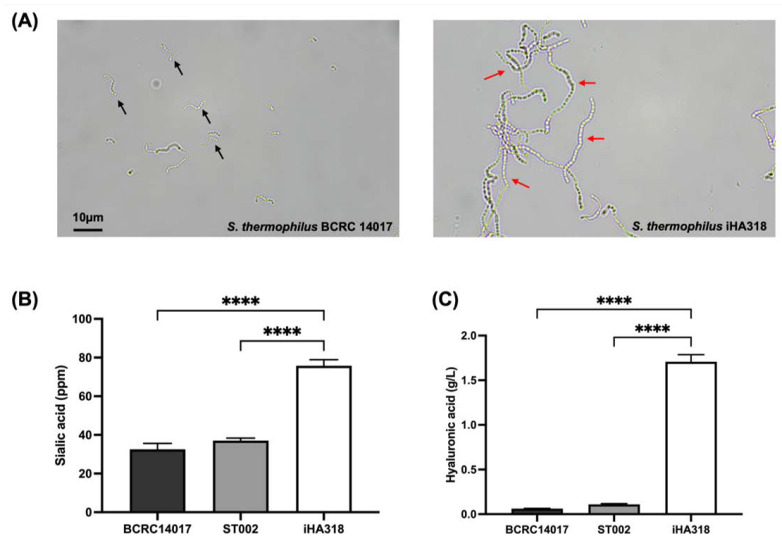
**Morphological and functional characterization of *S. thermophilus* strains.** (**A**) Microscopic images of *S. thermophilus* BCRC 14017 (**left**) and iHA318 (**right**) strains at 1000× magnification. Black arrows indicate BCRC 14017 strain, while red arrows point to iHA318 strain. Scale bar: 10 μm. (**B**) Sialic acid production by *S. thermophilus* strains BCRC14017, ST002, and iHA318. (**C**) Hyaluronic acid production by *S. thermophilus* strains BCRC14017, ST002, and iHA318. Data in (**B**,**C**) are presented as mean ± SEM. **** indicates a *p*-value < 0.0001.

All animal experiments were conducted according to the Institutional Animal Care and Use Committee guidelines of Chung Shan Medical University (IACUC No. 2709). Female ICR mice (6–8 weeks old) were purchased from BioLASCO Taiwan Co., Ltd (Taipei, Taiwan). The dry eye model was established by exposing mice to ultraviolet B (UVB) radiation daily from day 1 to day 7 at 0.72 J/cm^2^. The 11-day experiment consisted of an induction phase with UVB exposure and oral gavage administration of treatments from day −2 to day 7, as depicted in Figure 1. Mice were randomized into 5 groups (n = 5 per group): (1) control: 0.2 mL 0.9% saline without UVB; (2) damage: 0.2 mL 0.9% saline with UVB; (3) low-dose probiotics: low dose of *S. thermophilus* iHA318^®^ suspension (0.2 mL) with UVB; (4) high dose probiotics: high dose of *S. thermophilus* iHA318^®^ suspension (0.2 mL) with UVB; and (5) artificial tears: 0.2 mL 0.9% saline with artificial tear eye drops and UVB. Tear volume, tear film breakup time (TBUT), and ocular surface health assessments were performed on designated days. On day 8, the mice were euthanized after ocular evaluations for further analysis.

### 2.11. Tear Volume (Schirmer’s Test) and Tear Film Breakup Time (TBUT) Measurements

Schirmer’s test measured tear production according to the previously described method [30]. Briefly, a Tear Test Strip (Haag-Streit, Hertfordshire, UK) was gently placed in the lower eyelid pouch of each eye for 20 s. After that, the strips were removed and the wetted length was recorded in millimeters to quantify the tear volume. Tear film breakup time (TBUT) was measured as a standard diagnostic test for evaluating tear film stability in dry eye clinics [31]. This study modified the TBUT assessment procedure and used it in the mouse dry eye model. A small volume of sodium fluorescein dye solution (FLUO 900, Haag-Streit, UK) prepared in 0.9% NaCl saline was used. The fluorescein dye solution was applied onto the mouse ocular surface under broad cobalt blue illumination from a stereo fluoresce microscope (Zeiss Stemi SV11, Zeiss, Oberkochen, Germany). To spread the fluorescein dye evenly on the ocular surface, the examiner helped the mouse eye to blink a few times by gently massaging the eyelids. The mouse eye was then kept open for video-recording to determine the TBUT on a computer afterwards. The tear film breakup time (TBUT) was defined as the time interval in seconds between holding the eye open after the last blink and the first appearance of a dry spot or discontinuity in the fluorescein-stained tear film.

### 2.12. Ocular Surface Photography

According to a previously established method, corneal surface integrity was evaluated by grading several parameters, including smoothness, topography, opacity, and lissamine green staining extent [32]. For each mouse, one eye was randomly selected to assess corneal smoothness and topography, while the other eye was used for opacity grading. Digital images of the corneal surface were captured using a stereoscopic zoom microscope (SMZ 1500; Nikon Inc, Tokyo, Japan) equipped with a ring illuminator. Corneal smoothness was scored on a 5-point scale from 0 (no distortion) to 5 (severe distortion with no recognizable ring pattern) based on the number of distorted quadrants in the reflected ring: 1 = one quadrant, 2 = two quadrants, 3 = three quadrants, and 4 = all four quadrants distorted. Topography scoring used the same criteria but evaluated distortion over a larger corneal area. Opacity was graded from 0 (normal) to 4 (severe haze with ulceration) where: 0.5 = mild haze only visible under the dissection microscope, 1 = mild haze, 2 = moderate haze with visible iris, and 3 = severe haze with invisible iris. Both corneas were then stained with 2% lissamine green B and scored from 0 to 4 based on the extent of punctate staining and epithelial defects: 0 = punctate staining only, 1 = <25% with scattered punctate staining, 2 = 25–50% with diffuse punctate staining, 3 = 50–75% with punctate staining and defects, and 4 = >75% with abundant staining and large defects. All scoring was conducted on the captured images by two observers who were blinded to the experimental groups.

### 2.13. Statistics

This paper presents quantification data as mean ± standard error of the mean (SEM). Statistical analysis was conducted using GraphPad Prism software (Version 10.2.0). A non-parametric unpaired t-test (Mann–Whitney test) was utilized to compare two groups, while ANOVA with Dunnett’s multiple comparisons test was applied for analyses involving more than two groups. A *p*-value < 0.05 indicated statistical significance.

## 3. Results

### 3.1. Colony Characteristics and Bacterial Morphology

The *S. thermophilus* iHA318 strain was isolated from human breast milk and cultured under optimal conditions with the preferred nitrogen source. The iHA318 strain displayed typical streptococcal morphology, appearing as gram-positive cocci arranged in chains (Figure 1). Compared to the *S. thermophilus* BCRC 14017 strain, the colonies of iHA318 appeared larger and slightly raised with complete edges. In addition, other distinctions in morphology were observed (see Supplementary Figures S1 and S2, and Supplementary Tables S1 and S2). Notably, the length of the gram-positive cocci chains in the iHA318 strain was markedly longer compared to the BCRC 14017 strain (Figure 1A), suggesting potential variations in cell wall structure and organization between the two strains. The variations may affect their physiological and functional properties. The iHA318 strain also exhibited robust tolerance to acidic and bile salts conditions (see Supplementary Figure S3), indicating its resistance to gastrointestinal stresses. Additionally, compared with either *S. thermophilus* BCRC 14017 or ST002, which were two other strains isolated from human breast milk, iHA318 demonstrated significant secretion of sialic acid and hyaluronic acid (Figure 1B,C), implicating its potential as a probiotic with enhanced functional properties.

### 3.2. Protective Effects of S. thermophilus iHA318 on Human Retinal Pigment Epithelial Cells

Oxidative stress raised by reactive oxygen species (ROS), mainly generated from exposure to blue light and UV, is known as the major challenge to ocular cells, including the retinal pigment epithelium and photoreceptor cells [33]. Previous studies have shown that oxidative stress can damage the ocular surface and is closely linked to the mechanism of dry eye syndrome [3]. In addition, the cornea epithelial cells of dry eye patients are consistently damaged by elevated osmotic stress, which leads to inflammation and cell death, due to inadequate tear secretion [34].

**Figure 2:**
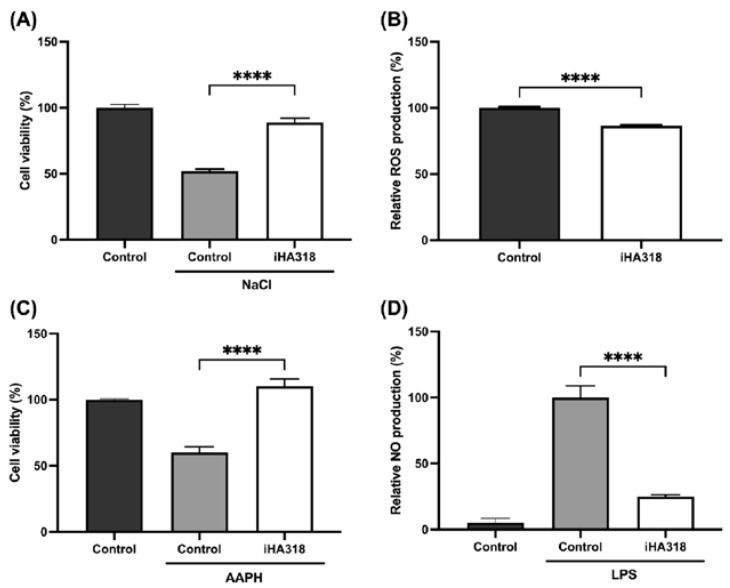
**Protective effects of *S. thermophilus* iHA318 on human retinal pigment epithelial cells (ARPE-19) and macrophages (RAW 264.7).** (**A**) Cell viability of ARPE-19 cells under osmotic stress induced by NaCl exposure, with or without iHA318 treatment. (**B**) Intracellular reactive oxygen species (ROS) production in ARPE-19 cells with or without iHA318 treatment. (**C**) Cell viability of RAW 264.7 macrophages exposed to oxidant AAPH, with or without iHA318 treatment. (**D**) Nitric oxide (NO) production by RAW 264.7 macrophages stimulated with lipopolysaccharide (LPS), with or without iHA318 treatment. Data are presented as mean ± SEM. **** indicates a *p*-value < 0.0001.

To investigate the potential protective effects of *S. thermophilus* iHA318 on ocular cells, the human retinal pigment epithelial cell line ARPE-19 was utilized as an example to assess its effects on osmoprotection and oxidative stress. Pretreatment with probiotics significantly increased ARPE-19 cell viability under high salt-induced osmotic stress conditions compared to cells subjected to osmotic stress without pretreatment (Figure 2A). In an evaluation assay of oxidative stress, ARPE-19 cells pretreated with *S. thermophilus* iHA318 exhibited substantially reduced intracellular reactive oxygen species (ROS) production compared to cells without probiotic administration (Figure 2B).

### 3.3. Immune Modulatory Effects of S. thermophilus iHA318

To further evaluate the antioxidant and anti-inflammatory properties of *S. thermophilus* iHA318, the murine macrophage cell line RAW264.7 was treated with the probiotic strain or the culture medium, followed by stimulation with the oxidant AAPH or the pro-inflammatory agent lipopolysaccharide (LPS), respectively. RAW 264.7 cells were chosen for this study due to their well-characterized inflammatory response, extensive use in similar studies investigating host–microbe interactions, and their ability to produce various inflammatory mediators, such as nitric oxide (NO), making them a suitable model for assessing the anti-inflammatory potential of probiotics [35,36,37]. The cells exposed to AAPH significantly exhibited significantly reduced cell viability compared to the untreated control. Remarkably, pretreatment with *S. thermophilus* iHA318 conferred substantial protection, enabling the experimental group to maintain a viability level comparable to that of the control after the AAPH challenge (Figure 2C). Similarly, pretreatment with *S. thermophilus* iHA318 significantly attenuated the LPS-induced inflammatory response, leading to a notable reduction in NO levels compared to those of the control group (Figure 2D). These results highlight the iHA318 strain’s immune-modulatory effects through alleviating oxidative stress and suppressing inflammation in macrophages.

### 3.4. In Vivo Effects of Probiotics on Tear Production

**Figure 3:**
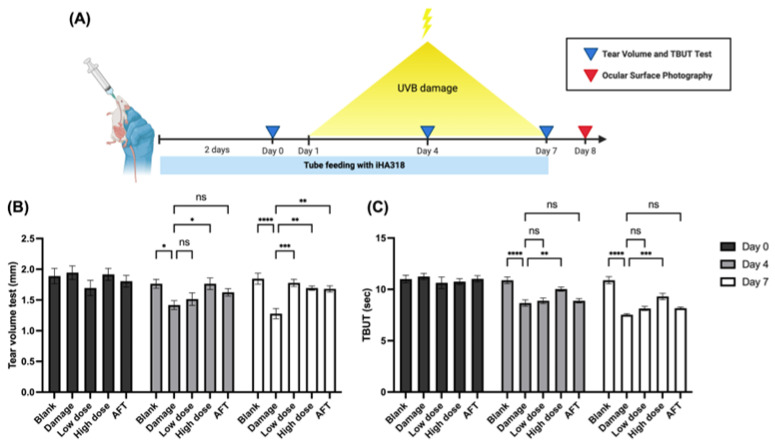
**Effects of oral administration of *S. thermophilus* iHA318 on tear volume and tear film stability in a mouse model of dry eye disease.** (**A**) Graphical illustration of the 11-day experimental timeline showing the periods of UVB exposure (days 1–7), oral gavage treatment administration (days −2 to 7), and evaluation time points for tear volume, tear film break-up time (TBUT), and ocular surface parameters. Blue triangles indicate tear volume and TBUT test days, while red triangles represent ocular surface photography days. (**B**) Tear volume measurements in mice from the blank, damage, low-dose iHA318, high-dose iHA318, and artificial tear groups on days 0, 4, and 7. (**C**) TBUT measurements in mice from the blank, damage, low-dose iHA318, high-dose iHA318, and artificial tear groups on days 0, 4, and 7. Data are presented as mean ± SEM. ns indicates no significant difference, * *p* < 0.05, ** *p* < 0.01, *** *p* < 0.001, **** *p* < 0.0001.

The dry eye model was induced by daily exposure to ultraviolet B (UVB) radiation with tear volume assessments conducted, following a treatment regimen spanning from day −2 to day 7 (Figure 3A). Initially, on day 0, no significant differences in tear volume were observed among the groups. However, on day 4, a marked increase was observed in the high dose probiotics group (1.764 ± 0.095) compared to the damage group (1.417 ± 0.071, *p* = 0.02). By day 7, all intervention groups demonstrated significantly higher tear volumes than the damage group (1.278 ± 0.082). Specifically, both the low dose probiotics group (1.778 ± 0.060, *p* = 0.0006) and high dose probiotics group (1.694 ± 0.035, *p* = 0.0051) exhibited substantial improvements (Figure 3B). These findings highlight the potential of probiotic intervention in enhancing tear production and suggest its therapeutic utility in managing conditions associated with tear deficiency.

### 3.5. Tear Film Break-Up Time (TBUT) Assessment

To understand whether the probiotics intervention could influence tear film stability, we conducted tear film break-up time (TBUT) assessments in the UVB-induced dry eye mouse model. No significant differences in TBUT were observed among the groups on day 0 (Figure 3C). However, on day 4, a notable increase in TBUT was observed in the high-dose probiotics group (10.030 ± 0.204, *p* = 0.0078) compared to the damage group (8.667 ± 0.301). Similarly, by day 7, the high dose probiotics group exhibited a significantly higher TBUT (9.306 ± 0.309) than the damage group (7.528 ± 0.100, *p* = 0.0003). These results indicate that high-dose probiotic intervention enhances tear film stability, suggesting its potential therapeutic efficacy in managing conditions associated with tear film instability and ocular surface dysfunction.

### 3.6. Ocular Surface Assessment

**Figure 4:**
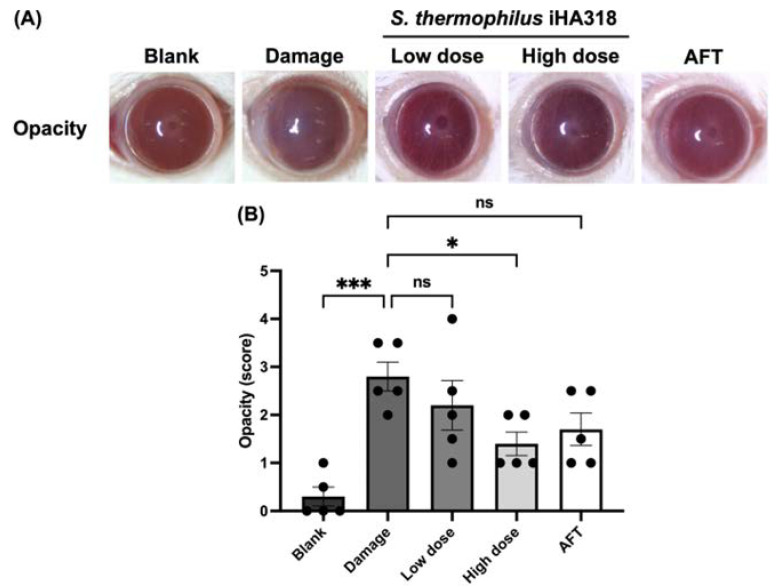
**Effects of *S. thermophilus* iHA318 on corneal opacity in a mouse model of dry eye disease.** (**A**) Representative images of corneal opacity in mice from the blank, damage, low-dose, high-dose, and artificial tear (AFT) groups on day 8. (**B**) Quantification of corneal opacity scores in mice from the blank, damage, low-dose iHA318, high-dose iHA318, and AFT groups on day 8. The opacity scores were determined by two observers who were blinded to the independent experiment (*n* = 5). Data are presented as mean ± SEM. ns indicates no significant difference; * indicates *p* < 0.05, and *** indicates *p* < 0.001.

**Figure 5:**
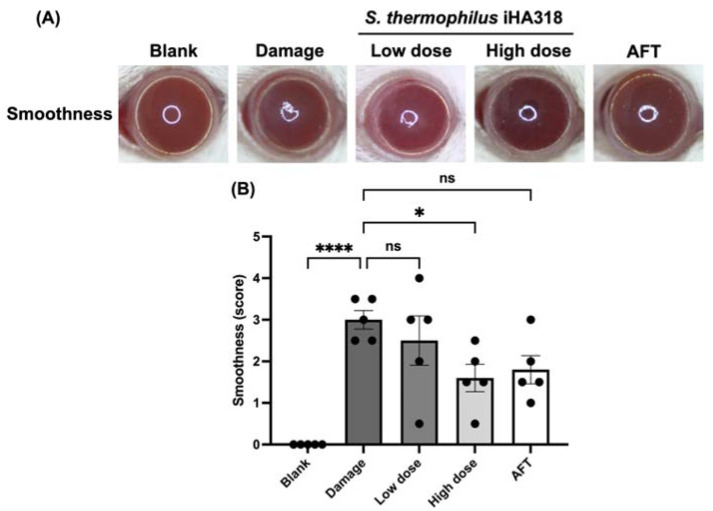
**Effects of *S. thermophilus* iHA318 on corneal smoothness in a mouse model of dry eye disease.** (**A**) Representative images of corneal smoothness in mice from the blank, damaged, low-dose iHA318, high-dose iHA318, and artificial tear (AFT) groups on day 8. (**B**) Quantification of corneal smoothness scores in mice from the blank, damage, low-dose, high-dose, and AFT groups on day 8. The smoothness scores were determined by two observers who were blinded to the independent experiment (*n* = 5). Data are presented as mean ± SEM. ns indicates no significant difference; * indicates *p* < 0.05, and **** indicates *p* < 0.0001.

**Figure 6:**
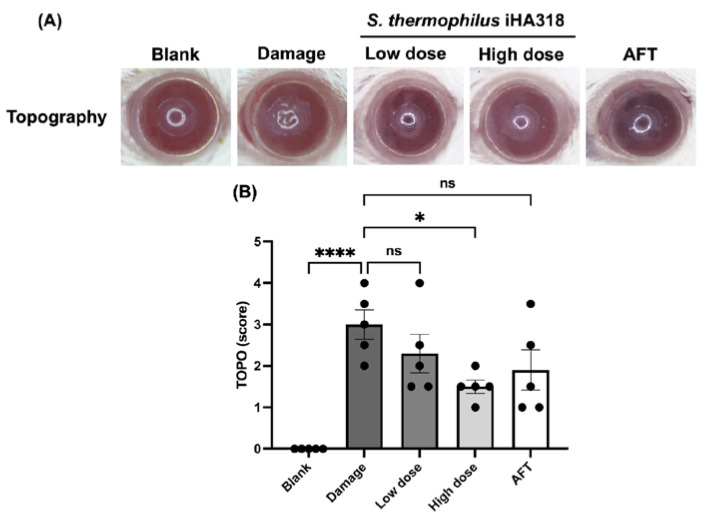
**Effects of *S. thermophilus* iHA318 on corneal topography in a mouse model of dry eye disease.** (**A**) Representative images of corneal topography assessment in mice from the blank, damage, low-dose iHA318, high-dose iHA318, and AFT groups on day 8. (**B**) Quantification of corneal topography scores in mice from the blank, damage, low-dose iHA318, high-dose iHA318, and AFT groups on day 8. The topography scores were determined by two observers who were blinded to the independent experiment (*n* = 5). Data are presented as mean ± SEM. ns indicates no significant difference; * indicates *p* < 0.05, and **** indicates *p* < 0.0001.

**Figure 7:**
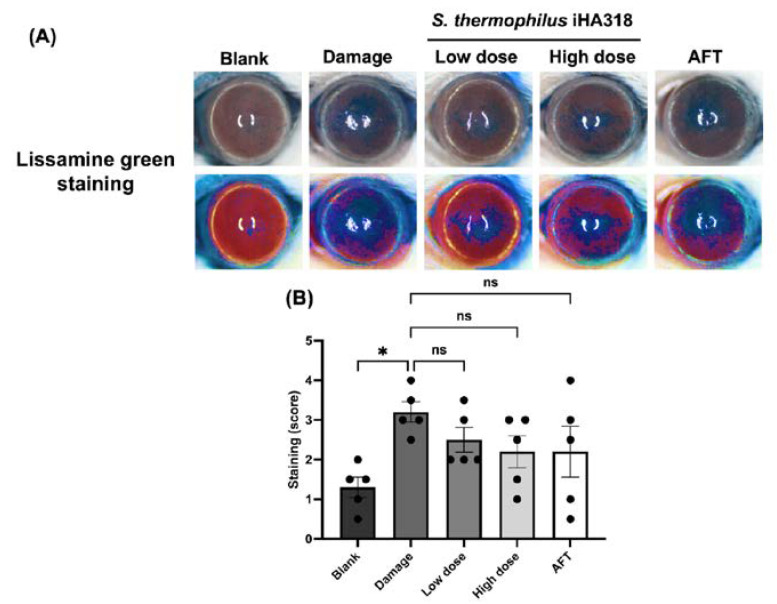
**Lissamine green staining evaluation of *S. thermophilus* iHA318 in a dry eye mouse model.** (**A**) Representative images of lissamine green staining on the ocular surface under different treatment conditions on day 8: blank, damage, low-dose iHA318, high-dose iHA318, and AFT. The upper row shows the original images, while the lower row shows the images with enhanced color temperature to better visualize the staining patterns. (**B**) Quantification of lissamine green staining scores by two blinded observers based on the independent experiments (*n* = 5). Data are presented as mean ± SEM. ns indicates no significant difference. * indicates *p* < 0.05.

Ocular surface health was evaluated through a series of assays including opacity, smoothness, topography (TOPO), and staining (lissamine green staining) in ICR mice [32]. These assessments have been used to elucidate the ocular surface parameters in a dry eye model induced by daily exposure to ultraviolet B (UVB) radiation [32]. Cornea opacity, a measure of corneal cloudiness, was reduced in the probiotics-treated groups in a dose-dependent manner (Figure 4). Particularly, the high-dose probiotics group (1.400 ± 0.245) exhibited a significantly lower opacity compared to the damage group (2.800 ± 0.300, *p* = 0.028). Similar trends were observed in the smoothness assay (Figure 5), where the high-dose probiotics group (1.600 ± 0.332) demonstrated significantly smoother ocular surfaces compared to the damage group (3.000 ± 0.224, *p* = 0.0367). Additionally, the topography assay showed consistent trends in Figure 6, with the high-dose probiotics group (1.500 ± 0.158) exhibiting significantly improved ocular surface topography compared to the damage group (3.000 ± 0.354, *p* = 0.021). However, in the staining assay, although no significant differences were observed between the treatment groups and the damage group, the degree of staining was reduced by probiotic treatment (see Figure 7). The overall findings suggest a potential protective effect of probiotic intervention against UVB-induced ocular surface damage, as evidenced by reduced opacity, improved smoothness, and better topography in the probiotics-treated groups.

## 4. Discussion

Dry eye is a pivotal and persistent challenge in global ocular health, has been a major concern in public health, and is regarded as a socio-economic burden. Most importantly, dry eye disease may lead to several severe eye disorders, such as chronic inflammation, infection, and corneal damage that may result in irreversible vision impairment [38]. However, a comprehensive and long-term solution for dry eye is still lacking and requires further investigation. Among the potential remedies, probiotics have emerged recently because of their anti-oxidative stress and anti-inflammatory characteristics. However, the hypothesized positive effects of probiotics to mitigate dry eye disease lack sufficient experimental evidence.

In the present study, we investigated the characteristic properties and therapeutic potential of *S. thermophilus* iHA318, focusing on ocular surface health, immune modulation, and tear production. Initial characterization revealed distinctive colony and bacterial morphology differences between *S. thermophilus* iHA318 and the BCRC 14017 strain, suggesting variations in cell wall structure that could impact functional properties. A previous study suggested that the *S. thermophilus* strains with longer cell chains have higher polysaccharide contents in the cell wall [39]. This feature may partially explain why the iHA318 confers a robust tolerance to acidic and bile salt conditions, indicative of its resilience, which is essential for exerting probiotic benefits in the gut. Moreover, comparative analyses highlighted increased secretion of sialic acid and hyaluronic acid by iHA318, suggesting its potential as a probiotic with superior functional attributes.

Dry eye patients encounter dual oxidative and osmotic stresses. Oxidative stress likely plays an initial role in inducing a dry eye status [3], which is then followed by increased osmotic stress due to insufficient tear secretion [34]. The concomitant occurrence of oxidative and osmotic stresses leads to chronic inflammation, typically observed in dry eye patients. Our results demonstrate that *S. thermophilus* iHA318 confers protective effects on human retinal pigment epithelial cells (ARPE-19) by providing osmoprotective and antioxidant properties. Pretreatment with iHA318 significantly enhanced cell viability under high salt-induced osmotic stress, and mitigated intracellular ROS production, suggesting a role in alleviating oxidative stress and maintaining cellular homeostasis. These findings, aligning with prior research results, have shown certain probiotic strains’ antioxidant and anti-inflammatory traits, contributing to protective effects against ocular surface damage and retinal degeneration [25,40,41,42].

Furthermore, iHA318 displayed potent immune-modulatory effects, preserving immune cell viability under oxidative stress and dampening inflammatory mediator release in macrophages. Macrophages play a crucial role in the immune response of the eye, acting as key regulators of inflammation and maintaining ocular surface homeostasis [43]. In dry eye syndrome, macrophages contribute to the pathogenesis through the production of pro-inflammatory cytokines and mediators, such as nitric oxide (NO) [2,3]. Dysregulation of macrophage function can lead to chronic inflammation and exacerbate the severity of dry eye syndrome [4]. Pretreatment with iHA318 enhanced cell viability after exposure to the oxidant AAPH and reduced nitric oxide (NO) secretion induced by the pro-inflammatory agent, lipopolysaccharide (LPS), in RAW 264.7 cells. This is a well-established in vitro model for studying macrophage function and inflammatory responses [35,36,37]. These results indicate modulating effects on immune responses through oxidative stress mitigation and inflammation suppression, which has potential implications for managing inflammatory conditions.

Moreover, in vivo studies in a dry eye mouse model revealed potent effects of probiotic intervention on tear production and tear film stability. Administration of iHA318 led to significantly higher tear volumes and tear film break-up time (TBUT), indicating enhanced tear production and stability, critical for ocular surface health maintenance. These findings support the therapeutic potential of probiotics in managing tear deficiency and instability-related ocular conditions [44]. Surprisingly, iHA318 is as effective as artificial tears in improving tear secretion.

Ocular surface assessments further supported the protective effects of probiotic intervention against UVB-induced ocular surface damage, with reduced corneal opacity, improved smoothness, and enhanced topography (indicating better surface regularity) observed in the probiotic-treated groups, indicating preservation of ocular surface integrity. Noticeably, iHA318 has been found to improve tear secretion and protect the ocular surface from damage. This suggests that iHA318 may offer a potentially better long-term alternative for dry eye treatment.

Current dry eye therapies, such as artificial tear supplements and anti-inflammatory agents, primarily aim to alleviate symptoms and provide temporary relief [45]. However, iHA318 presents a novel approach by targeting the underlying mechanisms of dry eye disease, including tear film instability, oxidative stress, and inflammation. Unlike artificial tears, which only provide temporary lubrication, iHA318 promotes the body’s natural tear production and enhances tear film quality through sialic acid and hyaluronic acid secretion. This approach addresses the root cause of tear film deficiency, potentially offering more sustained relief for dry eye patients. Moreover, while anti-inflammatory agents may help reduce ocular surface inflammation, they do not directly address the oxidative stress and hyperosmolarity contributing to dry eye pathogenesis. In contrast, iHA318 exhibits anti-inflammatory and cytoprotective effects, providing a multi-targeted approach to managing dry eye disease.

Despite the promising results, this study has certain limitations that should be acknowledged. First, the in vitro experiments were conducted using the ARPE-19 cell line, and RAW 264.7, which may not fully recapitulate the complex microenvironment of the ocular surface. Additionally, the in vivo study utilized a UVB-induced dry eye mouse model, which may not entirely reflect the multifactorial etiology of dry eye disease in humans. Furthermore, the study focused primarily on objective measures of dry eye disease, such as tear volume and ocular surface damage. It is crucial to evaluate the subjective symptoms and quality of life improvements in human clinical trials to assess the therapeutic potential of iHA318 fully.

Collectively, the rapid onset of action observed with iHA318 in improving tear volume and ocular surface health in the UVB-induced dry eye model highlights its potential for clinical applications. This probiotic strain could be incorporated into dry eye management protocols as a standalone therapy or combined with existing treatments. Future clinical trials are warranted to evaluate the efficacy, safety, and tolerability of iHA318 in human dry eye patients. Additionally, exploring the potential synergistic effects of iHA318 with other dry eye therapies may lead to more comprehensive and effective treatment strategies. In fact, we are currently preparing a follow-up study that includes histopathological analysis to further investigate the underlying tissue-level changes associated with *S. thermophilus* iHA318 treatment. This study will provide valuable insights into the mechanisms of action of iHA318 and its effects on the ocular surface at the cellular and tissue levels. We plan to submit this work for publication in the future, as it will complement the findings of the current study and contribute to a more comprehensive understanding of the therapeutic potential of iHA318. Furthermore, investigating the long-term effects of iHA318 on ocular surface health and exploring its potential applications in other ocular surface disorders, such as Meibomian gland dysfunction [46] or ocular surface disease associated with systemic conditions [47], could broaden its therapeutic scope.

## 5. Conclusions

In conclusion, our findings underscore the multifaceted therapeutic effects of *S. thermophilus* iHA318 in modulating immune responses, enhancing tear production, and maintaining ocular surface health. Further elucidation of the underlying mechanisms and optimization of probiotic interventions are warranted for ocular health maintenance and disease management.

## Figures and Tables

**Table 1:** Primers Used for PCR Amplification.

Group	Universal Primer	Sequences
1	27F	AGAGTTTGATCMTGGCTCAG
	1525R	AAGGAGGTGWTCCARCC
2	8F2	TGGAGAGTTTGATCCTGGCTCAG
	806R	GGACTACCAGGGTATCTAAT
3	fD1 mod	AGAGTTTGATCYTGGYTYAG
	16S1RR-B	CTTTACGCCCARTRAWTCCG

## Data Availability

The data presented in this study are available on request from the corresponding author.

## Supplementary Materials

The following supporting information can be downloaded at: https://www.mdpi.com/article/10.3390/microorganisms12071306/s1, Figure S1: Cell count of S. thermophilus iHA318 cultured in the MRS medium with or without supplements; Figure S2: Cell count of S. thermophilus iHA318 cultured in the MRS medium with varied urea concentration; Figure S3: Acid and Bile Salt Tolerance of S. thermophilus iHA318. Table S1: Basal culture media composition with varied nitrogen and carbohydrate supplements; Table S2: Basal culture media composition with varied nitrogen concentration.
