# Supplementary material for: Streptococcus thermophilus iHA318 Improves Dry Eye Symptoms by Mitigating Ocular Surface Damage in a Mouse Model
Source: Microorganisms. 2024 Jun 27;12(7):1306. doi: 10.3390/microorganisms12071306 (PMC11279365; doi:10.3390/microorganisms12071306)
Supplement: Supplementary file 1 [file microorganisms-12-01306-s001.zip › Supplementary Information.pdf]

Supplementary Information

Streptococcus thermophilus iHA318 Improves Dry Eye Symptoms through Mitigating Ocular Surface Damage in a Mouse Model

Optimization of Supplementary Source for *Streptococcus thermophilus* iHA318

To determine the optimal supplementary source for culturing the *S. thermophilus* iHA318 strain, bacterial growth was evaluated in media formulations containing different nitrogen and carbohydrate compounds. The *S. thermophilus* iHA318 strain was cultured in media A-I (listed in Table 1), which were identical except for the 5 g nitrogen source and 5% carbohydrate source component: (A) no nitrogen source, (B) skimmed milk powder, (C) tryptic soy, (D) urea, (E) meat extract, (F) yeast extract, (G) casein, (H) lactose, and (I) fructose. Following 24 hour anaerobic incubation at 37°C, bacterial counts were determined by pour plating on MRS agar.

Table 1. Basal culture media composition with varied nitrogen and carbohydrate supplements.

Medium group	A	B	C	D	E	F	G	H	I
Supplement	none	skimmed milk powder (5 g)	tryptic soy (5 g)	Urea (5 g)	meat extract (5 g)	yeast extract (5 g)	Casein (5 g)	Lactose (50 g)	Fructose (50 g)
Basal media composition	Peptone (10 g), beef extract (10 g), yeast extract (5 g), Magnesium Sulfate (0.1 g), dextrose (20 g), Polysorbate 80 (1 g), Ammonium Citrate (2 g), Sodium Acetate (5 g), Manganese Sulfate (0.05 g), and Dipotassium Phosphate (2 g)								
H_2O	Quantify to 1 liter								

As shown in **Figure 1**, inclusion of urea as the nitrogen source in medium D resulted in a significant increase in bacterial growth compared to the no nitrogen source control (medium A). The other nitrogen sources, including skimmed milk, tryptic soy, meat

extract, and casein supported only modest increases in growth. Notably, yeast extract (medium F), and carbohydrate sources (medium H and I) led to a reduction in growth of the iHA318 strain compared to the no nitrogen source control. These results indicate that small molecular nitrogen sources, such as urea, are more optimal for culturing *S. thermophilus* iHA318 than larger macromolecular nitrogen sources like protein hydrolysates or extracts.

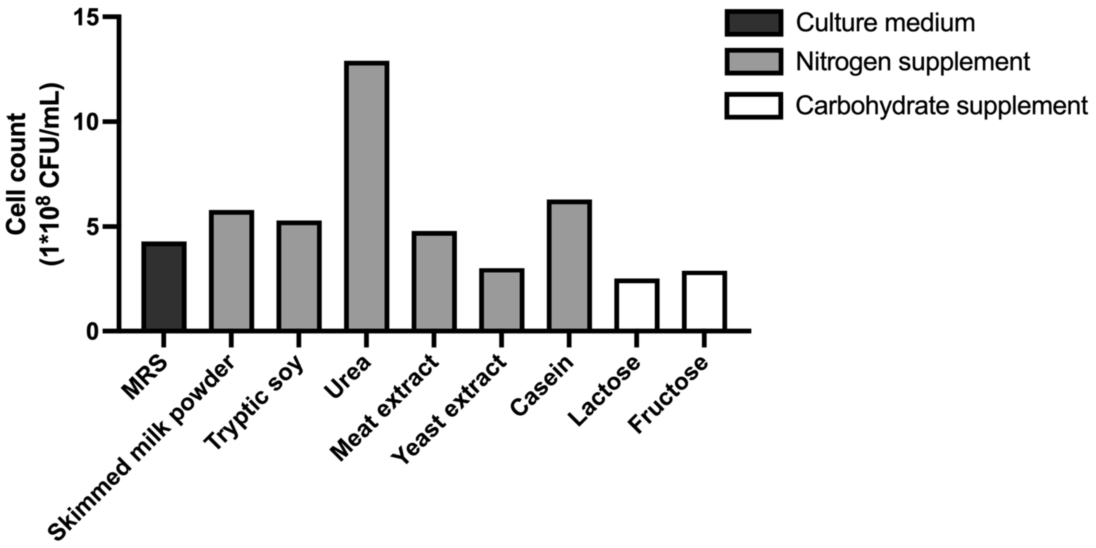

Supplementary Figure 1. Cell count of *S. thermophilus* iHA318 cultured in the MRS medium with or without supplements.

Effect of different supplements (nitrogen sources or carbohydrate sources) on the growth of *S. thermophilus* iHA318. Bacterial cultures were grown anaerobically at 37°C for 24 hours in basal media supplemented with 5 g/L of the indicated nitrogen source: skimmed milk powder, tryptic soy, urea, meat extract, yeast extract, or casein; 50 g/L of carbohydrate sources: lactose and fructose. A basal medium control was also included. After incubation, bacterial cell counts are determined and reported as 10^8 CFU/mL. (n=1)

Optimization of Urea Concentration

To further optimize the nitrogen source concentration, growth of *S. thermophilus* iHA318 was assessed in medium D formulations with varying urea levels from 0 - 1% w/v (media D-1 to D-4, Table 2). Following 24 hour anaerobic incubation at 37°C,

bacterial counts were determined by pour plating on MRS agar.

Table 2. Basal culture media composition with varied nitrogen concentration.

Medium group	D-1	D-2	D-3	D-4
Urea (concentration)	0 g (0 %)	1 g (0.1 %)	5 g (0.5 %)	10 g (1.0 %)
Basal media composition	Peptone (10 g), beef extract (10 g), yeast extract (5 g), Magnesium Sulfate (0.1 g), dextrose (20 g), Polysorbate 80 (1 g), Ammonium Citrate (2 g), Sodium Acetate (5 g), Manganese Sulfate (0.05 g), and Dipotassium Phosphate (2 g)			
H_2O	Quantify to 1 liter			

As depicted in **Figure 2**, inclusion of 0.5% urea (medium D-3) led to an obvious increase in iHA318 growth compared to the no urea control (D-1). Higher urea concentrations of 1% (D-4) did not further enhance growth, while 0.1% urea (D-2) provided no appreciable growth benefit over the no urea condition.

Collectively, these results indicate that 0.5% urea represents the optimal nitrogen source and concentration for achieving maximal growth of the *S. thermophilus* iHA318 strain in this basal medium formulation. This optimized urea-supplemented medium was used for all subsequent experiments with the iHA318 strain.

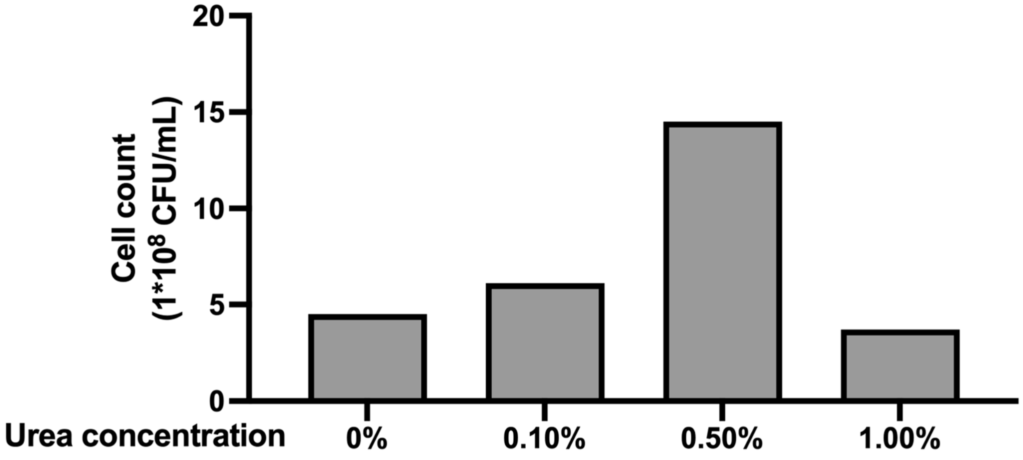

Supplementary Figure 2. Cell count of *S. thermophilus* iHA318 cultured in the MRS medium with varied urea concentration.

Effect of urea concentration on the growth of *S. thermophilus* iHA318. Bacterial

cultures were grown anaerobically at 37°C for 24 hours in basal media supplemented with 0%, 0.1%, 0.5%, or 1% urea as the nitrogen source. After incubation, bacterial cell counts are determined by MRS pour culture assay and reported as 10^8 CFU/mL. (n=1)

Acid and Bile Salt Tolerance of *S. thermophilus* iHA318

The ability of the *S. thermophilus* iHA318 strain to tolerate acid and bile salt stress conditions was assessed. For acid tolerance testing, 10 mg of iHA318 powder was inoculated into MRS broth adjusted to pH 3.0 with HCl. Bile salt tolerance was evaluated by supplementing MRS with 0.3% bile salts. Probiotic cultures were incubated at 37°C for 4 hours, after which cultures were serially diluted in PBS and plated onto MRS agar for CFU counting. Plates were incubated anaerobically at 37°C for 72-96 hours before evaluating bacterial colonies. Cell counts were calculated and expressed as log CFU/mL values. As shown in **Figure 3**, after 4 hours of exposure to pH 3.0 or 0.3% bile salt conditions, the iHA318 strain exhibited equivalent growth compared to the control cultures (0 hr). These results demonstrate that *S. thermophilus* iHA318 can effectively tolerate and maintain viability under highly acidic pH 3.0 conditions as well as in the presence of 0.3% bile salts over 4 hours of exposure. The ability to withstand these gastrointestinal tract challenges suggests iHA318 may be capable of surviving passage through the stomach and small intestine to potentially confer probiotic benefits.

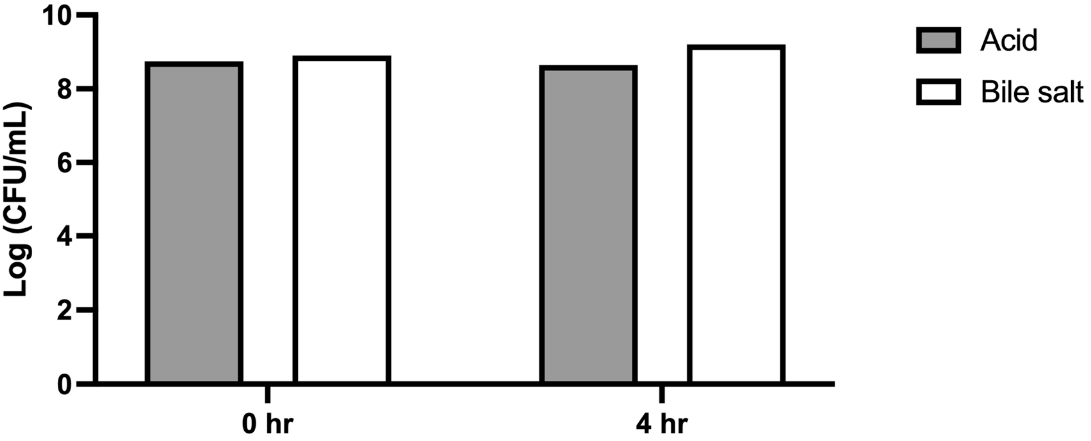

Supplementary Figure 3. Acid and Bile Salt Tolerance of *S. thermophilus* iHA318

Cultures were inoculated with 10 mg of iHA318 powder into MRS broth adjusted to pH 3.0 with HCl (acid tolerance) or supplemented with 0.3% bile salts. After 0 and 4 hour incubations at 37°C, cultures were serially diluted and plated on MRS agar for 72-96

hour anaerobic incubation. Cell count of iHA318 in acidic pH 3.0 (gray bar) or 0.3% bile salts (white bar) conditions expressed as log CFU/g. (n=1)
