# Supplementary figures and images for: Streptococcus thermophilus iHA318 Improves Dry Eye Symptoms by Mitigating Ocular Surface Damage in a Mouse Model
Source: Microorganisms. 2024 Jun 27;12(7):1306. doi: 10.3390/microorganisms12071306 (PMC11279365; doi:10.3390/microorganisms12071306)

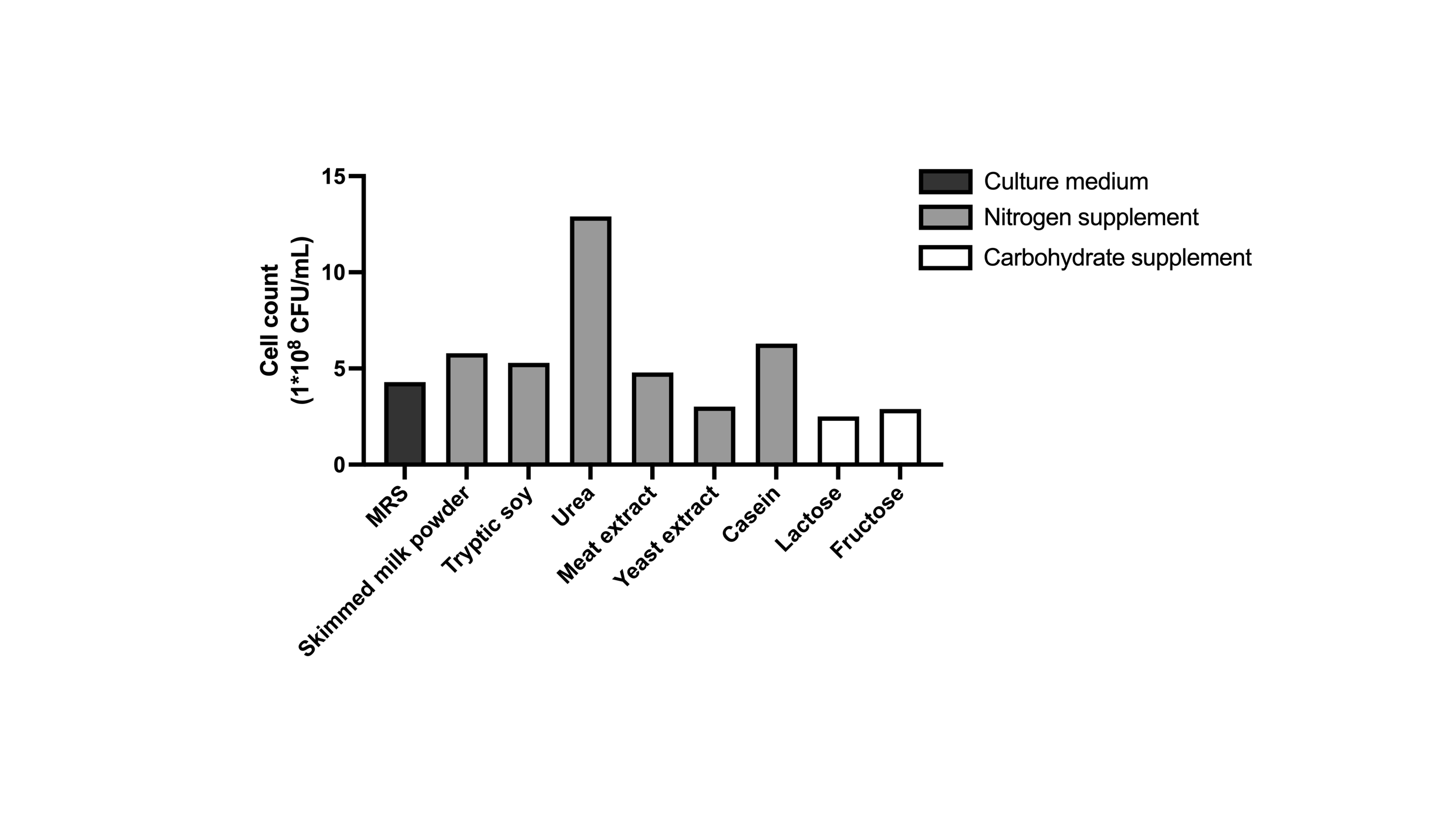

Supplement: Supplementary file 1 [file microorganisms-12-01306-s001.zip › sFigure 1.tiff]

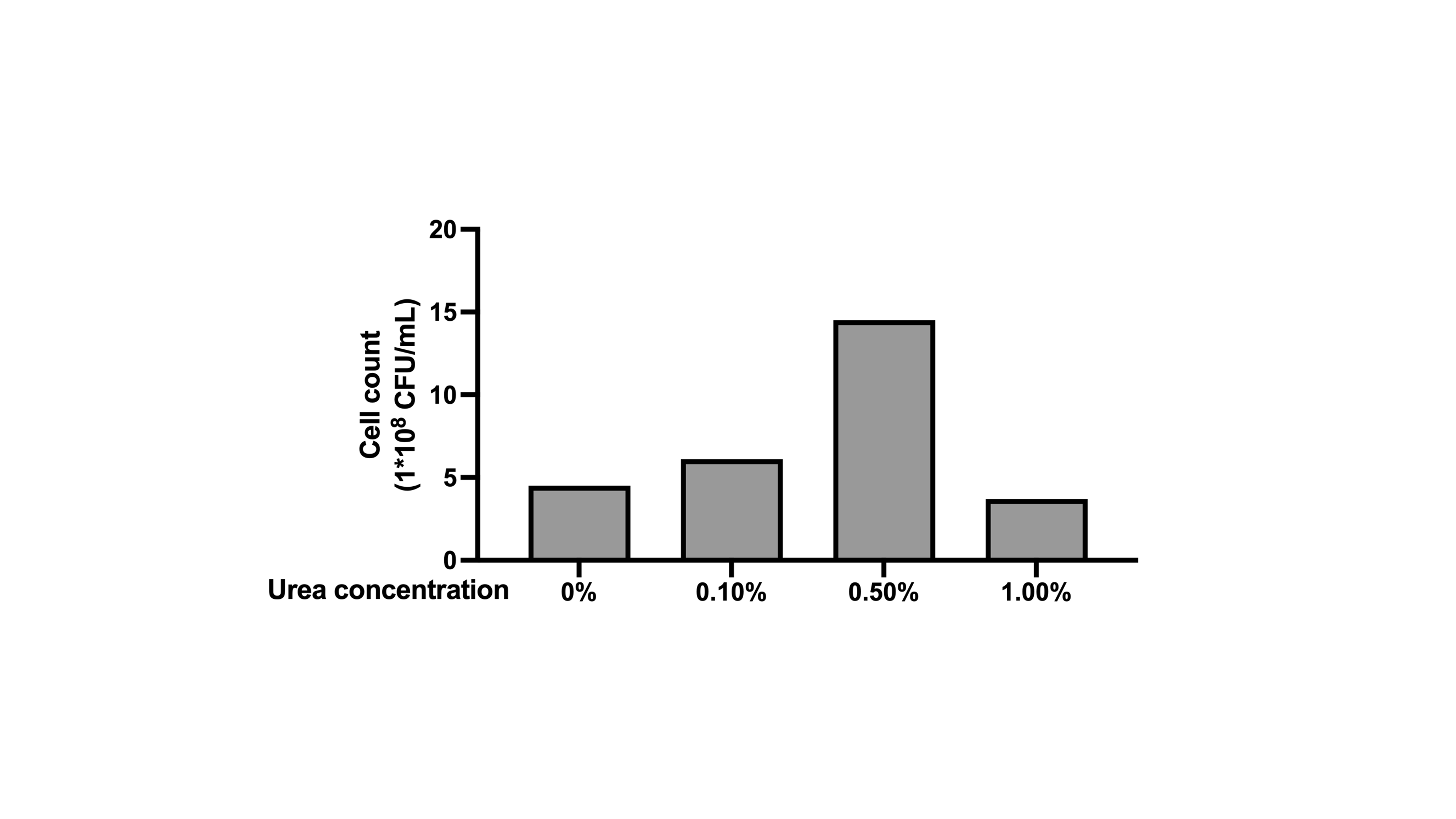

Supplement: Supplementary file 1 [file microorganisms-12-01306-s001.zip › sFigure 2.tiff]

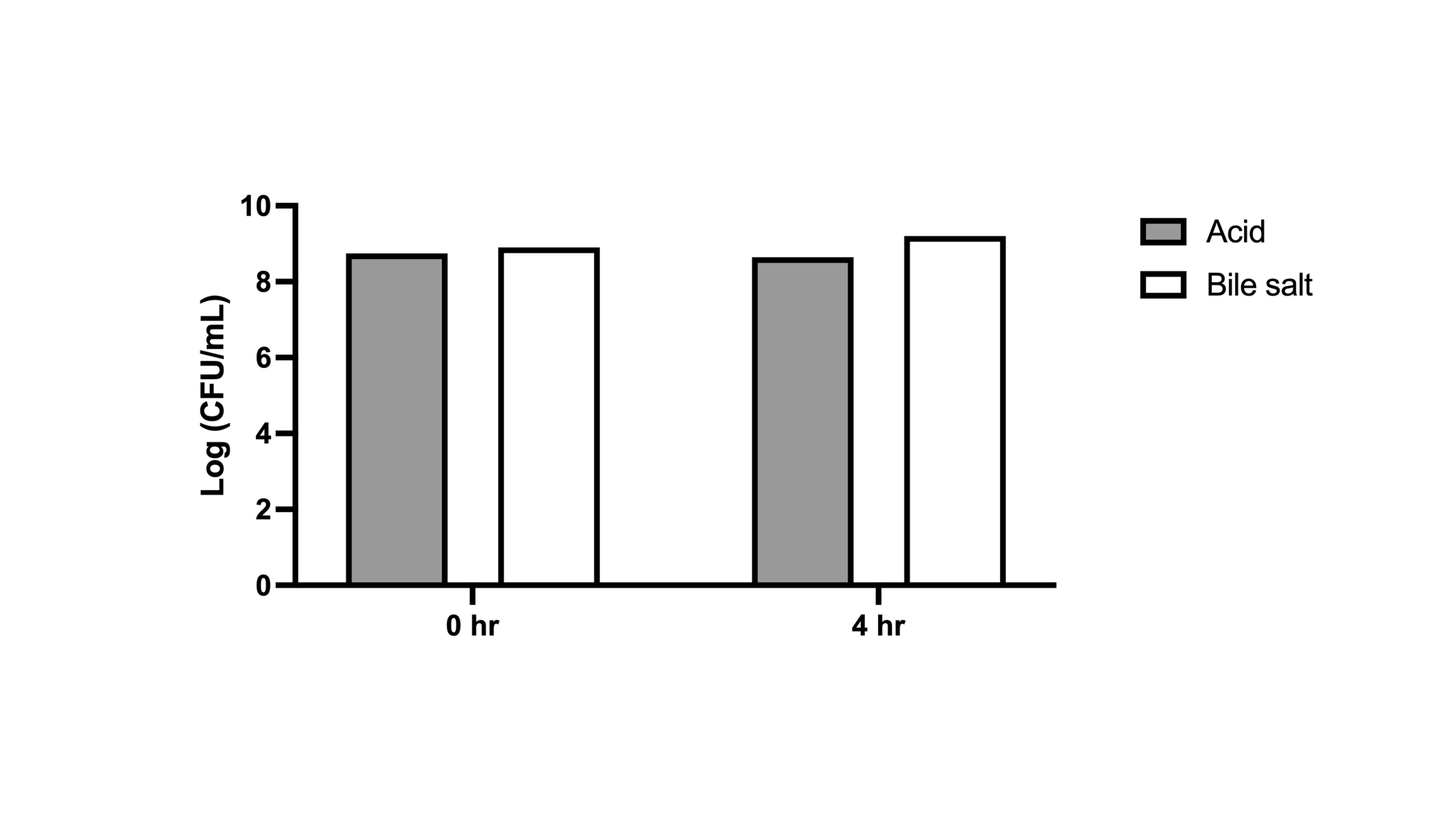

Supplement: Supplementary file 1 [file microorganisms-12-01306-s001.zip › sFigure 3.tiff]
